# Contemporary and Future Development of 3D Printing Technology in the Field of Assistive Technology, Orthotics and Prosthetics

**DOI:** 10.33137/cpoj.v6i2.42225

**Published:** 2023-12-22

**Authors:** Beygi B Hassan, M.S. Wong

**Affiliations:** The Department of Biomedical Engineering, The Hong Kong Polytechnic University, Hong Kong.

**Keywords:** 3D Printing, Orthotics, Prosthetics, Adolescent Idiopathic Scoliosis, Rehabilitation, Digitalization, CAD/CAM, Technology, Additive Manufacturing

## Abstract

3D printing is considered as a helpful technology that facilitates innovative assistive technology, orthotics, and prosthetics development. This technology could likely contribute to positive treatment outcomes. It could also mitigate the challenges encountered when using the traditional methods. Our team's research in the application of 3D printing in prosthetics, orthotics and biomedical technology has shown beneficial results in its use. This article gives a general description on application of CAD/CAM, digitalization and 3d printing in this industry followed by short description of two spinal-related projects conducted in our research team. Technological and clinical challenges on utilization of this technology have been listed. Finally, this manuscript provides recommendation for broader applications and developments of the aforementioned technology through interdisciplinary practices. A glimpse into the future of 3D printing in the healthcare industry shows that this industry is poised to continue having a significant impact in this sector. It should be emphasized that assistive technology, orthotics, and prosthetics require a human touch and connection, and no digital tool or technology can replace such requirements. Indeed, multi-disciplinary collaboration is the key to the success of applications of 3D printing.

## INTRODUCTION

Three-dimensional (3D) printing is an additive manufacturing process to make 3D objects. With the evolution of this technique since the 1980s, its applications are becoming increasingly relevant to users of assistive technology, orthotics, and prosthetics services. To be more precise, we believe this digital transformation should be looked as a subset of more comprehensive technology, CAD/CAM (Computer-aided Design/Computer-aided Manufacturing). The CAD software assists clinicians in capturing the shape of body segment(s) and designing purposeful devices for treatment purposes. It achieves this by reading a 3D captured shape of body segment from advanced 3D scanners and allowing for modifications to be made to the limb shape. The CAM facilitates in the manufacturing process with reduction of manpower and increase of precision. 3D printing is only a part of this digital transformation, referring only to the additive manufacturing process, which is available in different types and forms, including powder, liquid, and solid materials.^1^

Clinical and technological advancements are equally important in delivering quality assistive technology, orthotics, and prosthetics services. Patients should be comprehensively assessed before an appropriate prescription of assistive technology, orthosis, or prosthesis is given. In the fabrication of assistive technology, orthosis, or prosthesis, advanced technological developments have the potential to offer the end-users an effective and reliable service. However, the enhancement of CAD/CAM (3D printing) technology should focus on facilitating the relevant clinical treatment outcomes.

3D printing serves as a revolutionary transformation in the design and fabrication of orthotic and prosthetic devices, reshaping the landscape with an excellent potential to improve the lives of people with disabilities. This technology could be deployed for use in hybrid manufacturing together with subtractive manufacturing or injection molding processes to produce a standalone 3D-printed appliance or sophisticated system. Depending on the size and complexity of the design, it can either produce a whole shape through the printing process or print the parts which could be assembled to complete the system.

The materials currently used to design and fabricate 3D printed orthotic and prosthetic devices include ABS, Nylon (PA 12 and PA 11), PETG, PP, TPU, fiber reinforced composites, and silicone resins. There are several advantages to using 3D printing, including, but not limited to:^2–7^

- Material savings with less waste (plaster or foam blanks) therefore being more environmentally friendly.
- Elimination of storage of hard copy models, which saves space.
- Reduction of production time, labor-intensive manual work, and cost. For example, the lower-cost 3D printed prosthetics compared to expensive antiquated traditional prosthetics in which artisanal approaches are deployed.
- Solutions of customization and personalization as complicated designs could be easily implemented, a fact that can not be achieved with traditional manufacturing (thermoforming and lamination). For example, integrating unique patterns, personalized textures, variations in thickness across different areas, and incorporating built-in reinforcement profiles into the designs enhances the durability and breathability of the device. This approach likely promotes the use of prescribed orthotic or prosthetic devices among patients.
- Decrease of weight, as compared to traditional manufacturing, in which the plastic sheets or laminated layers were made as solid components, the implementation of hollow inside the layers by optimizing the infill to strike a balance between weight, strength, and printing time of the orthotic and prosthetic components. Furthermore, a lattice structure could be implemented to decrease the weight and provide shock absorption properties, for example, in insoles.
- Extending the reach of 3D-printed orthotic and prosthetic devices to remote areas as well as less developed countries.
- Ease of addressing the continued growth of the children.

The above-mentioned advantages could facilitate a wide range of devices and applications in the orthotics, prosthetics and assistive technology including:

- Inner and outer sockets for lower limb and upper limb loss, as well as cosmetic covers and hand prostheses
- Wheelchair accessories, including cup holders, custom wheelchair seats, adaptive utensils with enlarged grips, or customized shapes to help people with motor disabilities enhance feed.
- Customized AFOs, cranial remodeling helmets, insoles and footwear, hand splints, and spinal orthoses.
- Customized seating and positioning systems enhance the sitting posture of people with disabilities to decrease the chance of pressure sores.

## OUR TEAM'S EXPERIENCES IN THIS AREA

In considering the numerous advantages, our team has carried out research exploring the treatment effectiveness of the CAD/CAM method as compared to manual plaster casting method in managing adolescent idiopathic scoliosis. In the first study, the clinical parameters of Cobb angle and apical vertebral rotation were evaluated at the pre-orthosis and the initial in-orthosis visits of 40 subjects. The mean decrease of Cobb angle was almost 10% higher for the CAD/CAM method. The mean rectification time of the CAD/CAM method was shorter than that of the conventional manual plaster modification. Our conclusion was that the CAD/CAM method can provide similar clinical results in the initial stage of the treatment compared to the manual method while saving time in rectification by 108.3 minutes (63.5%).^8–9^

In the second study, our team conducted a prospective randomized controlled trial to compare **1)** clinical effectiveness and **2)** quality of life between 3-D printed spinal orthoses and conventional orthoses. The conventional orthoses were made using vacuum forming technique to drape the heated plastic sheet on a positive trunk model. The study was limited to adolescent idiopathic scoliosis (AIS). Thirty females with AIS who met the inclusion criteria (age 10–14, Cobb angle 20–40°, Risser sign “skeletal maturity” 0–2, and less than one year after the first menses) were recruited. Patients were excluded if they suffered from musculoskeletal or developmental disease that prevented them to be complied with the treatment protocol. Those recruited were randomly allocated to either 3D printing or conventional group. 3D printing technology was used to design and fabricate orthoses to manage AIS, aiming to improve in-orthosis correction and patient compliance. 3D printed orthoses were made with 2.5 mm thick Nylon 12 (PA–12) using the Stratasys machine and FDM technique. The result showed that the patients wearing the 3D-printed orthoses experienced better clinical outcomes than those in the control group (conventional orthotic design) in terms of quality of life (QoL) measured by questionnaire as well as similar immediate in-orthosis correction. Cobb angle in 3D printing orthosis group decreased from 31.7°±6.0 in the baseline to 19.4°±3.9 in immediate in-orthosis condition. In the conventional orthosis fabrication group, the Cobb angle of 29.8°±4.4 in the baseline showed a decrease to reach to in-orthosis Cobb angle of 16.5°±6.8.^10^ It is proposed for future studies to consider reinforcement in strategic force application regions of the spinal orthosis using variant thickness in the different areas as well as exploring other materials and printing technologies that could be used for spinal bracing, e.g., polypropylene filament (PP) which has been deployed in some clinics recently.

Our experiences and findings in this study have led us to believe this technology has wider applications than traditional orthotic and prosthetic practice. For example, it can be deployed in assistive technology for animals who have lost limbs, fractures, or deformed limbs can access better medical care through 3D printing technology with customized artificial limbs, braces, pads, beaks, etc., which could increase their quality of life and longevity.

Another example of potential application of this technology is work we have done beyond the recently defined scopes of services for orthotists and prosthetists now deployed in Hong Kong for several years, is the provision of patient-specific surgical guides for intra-operative application and anatomical models to assist doctors in pre-operative planning through the application of 3D printing technology.

Irrespective of application, one of our findings across these various fields is that innovative technology application in the clinical setting could be only helpful and valuable when a team of experts with different backgrounds work together and supplement each other in both the development and application stages. The application of CAD/CAM (3D printing) technology could range from simple to complex assistive technology, orthosis, or prosthesis in which a wide range of clinical and technological knowledge, skills, and experiences is envisaged and needed. This can only be done with close collaboration among professionals from different disciplines.

In working together interdisciplinarity, we have identified two general areas of importance that need to be considered by other researchers working on this **1)** the technological challenges and **2)** the clinical challenges.

## TECHNOLOGICAL CHALLENGES

There are several technological challenges, such as the selection of appropriate printing method, i.e., FDM (Fused Deposition Modeling), SLS (Selective Laser Sintering), SAF (Selective Absorption Fusion), SLA (stereolithography) or MJF (Multi Jet Fusion) for the specific clinical purpose(s) as we also faced with some of them upon our clinical studies. Considering the product allowance in terms of dimension, material, or bonding properties is essential. Some vital physical and mechanical properties tests for suitability of clinical applications should be included, such as tensile, bending, and opening-closing tests, as well as body heat and humidity tests within the laboratory.

The settings of 3D printing machines are versatile. For FDM, it includes the speed of 3D printing, the size and temperature of the nozzle, and the bed temperature, which may all affect the adhesion/bonding of the materials. On the other hand, the limited number of materials in SLS technology, as well as powder quality, distribution, and density, play a vital role in the quality of 3D printed products. Therefore, sometimes, the design needs to proceed using a trial-and-error process to obtain the desired orthotic or prosthetic component.

Another challenge is the limited selection of materials, as the material and the final orthotic or prosthetic device should pass a biocompatibility test to be considered medical-grade 3D printing materials. Materials must meet ISO standards regarding toxicity, skin irritation, and sensitization. The biggest challenge arises from assessing how materials react with the skin over the long term, as orthotic or prosthetic devices maintain direct contact with the skin.

Improvement of the surface finishing and durability of 3D printed prosthetics is another crucial element as FDM technology leaves distinct layer lines, and SLS and MJF make a matte surface. Post-process surface finishing is either physical smoothing, like sandblasting, or chemical smoothing, such as vapor smoothing through vaporized solvents.

Flexible printing materials are in demand in the O&P industry, and the most acceptable material up to this point of time tends to be TPU. However, it faces considerable difficulty because of its durability issue. As such, the vapor smoothing improves durability. To address bacteria proliferation, particularly on surfaces directly in contact with skin, such as liners, surface finishing could protect any liquid intake. Nonetheless, this needs further improvement and accessibility to clinics and laboratories worldwide.

Finally, it is worth noting that in the application of additive manufacturing, several CAD and 3D printing software requite a continued internet connection to identify license eligibility. While this requirement is understandable, alternative methods must be considered, as offline platforms are vital for those working in remote areas and providing orthotic and prosthetic services. Application of generic Mesh modification software in particular freeware versions could be kept into account.

## CLINICAL CHALLENGES

There are a number of clinical challenges, such as material allergies, the effect of body temperature and humidity on the materials used, and short-term and long-term clinical studies on acceptance and efficacy. As an analogy to the conventionally used thermoplastics, the 3D printed materials should be easily handled, including machining, grinding, and possibly modifying the 3D printed devices using the heat gun.

One of the biggest challenges lies in the mindset of clinicians and technicians, who may be hesitant to partially transition from conventional methods to embracing digitalized 3D printing. Overcoming this reluctance requires them to be open to learning and adapting themselves to become proficient digital practitioners. Therefore, the re-education of practitioners should be taken into consideration, as it would require the acquisition of new skills. Nonetheless, it is essential to acknowledge that the expertise and creativity of clinicians and designers in utilizing digital tools within 3D design applications are critical. These skills can be transformed from traditional methods and effectively implemented in digital formats. It also reminds us the necessity to incorporate more components into the curriculum in different universities to synchronize with the rapid advancement in digital technologies. This will prepare students to be already comfortable and proficient with such technology once they start the clinical services.

## FUTURE OUTLOOK OF 3D PRINTING LANDSCAPE

The market is expected to become more populated with machine learning and AI (artificial intelligence) models and semi-automated designs through advancements in software, 3D scanning, and digitalized 3D printing workflow. While it is imperative for clinicians to get familiar with digital design, a semi-automated, repeatable workflow will help clinicians spend more time improving the designs, rather than grappling with digital tools to replicate traditional designs.

More 3D printing machines will be anticipated into the orthotic and prosthetic market while 3D printing hardware, software, and materials will evolve concurrently.

The development of new materials will provide further options in the selection of preferred materials for orthotic and prosthetic devices. The new materials could also address the need for durability, for example, in a remote, hot, and humid climate where the maintenance and frequent follow-up to the fitted devices may not be feasible. The application of pellet 3D printing could be considered as one of the solutions since there are a variety of thermoplastic materials in the form of pellets compared to the currently limited options for the filaments in the market.

The application of metal 3D printing may get widespread, particularly for custom-made prosthetic and orthotic components for athletes or activity-specific devices. Currently, these components are mainly manufactured using subtractive manufacturing, including CNC and laser cutting. However, once the metal 3D printing machines become more affordable, they could help clinicians design and manufacture the specific components tailor-made for the individual amputees.

Color printing is another domain that can be further practiced. It remarkably improves the adherence of children with disabilities/deformities to the prescribed orthotic/prosthetic appliances thanks to its enhanced aesthetic. Like metal 3D printing, this is pending the development of more cost-effective machines in the near future.

In summary, 3D printing would become a helpful technology that facilitates innovative assistive technology, orthotics, and prosthetics development. This technology could likely contribute to positive treatment outcomes. It could also mitigate the challenges encountered when using the traditional methods. However, it should be emphasized that assistive technology, orthotics, and prosthetics require a human touch and connection, and no digital tool or technology can replace such requirements. Indeed, multi-disciplinary collaboration is the key to the success of applications of 3D printing.

## CALL TO ACTION

Close collaboration among different disciplines is the essential prerequisite for the successful application of such technology into the clinical setting. Moreover, a number of relevant clinical and technical research studies should be conducted before the CAD/CAM (3D printing) technology can be established and formulated as an evidence-based patient-centered practice in the field of assistive technology, orthotics, and prosthetics. The patient's safety should be listed as a priority in the implementation of this technology.

## DECLARATION OF CONFLICTING INTERESTS

Authors declare that there is no financial or personal relationship with organizations or individuals that might have influenced our research.

## AUTHORS CONTRIBUTION

Both authors contributed equally to the research and the writing of this manuscript.

## SOURCES OF SUPPORT

None.

## AUTHORS SCIENTIFIC BIOGRAPHY

**Figure:**
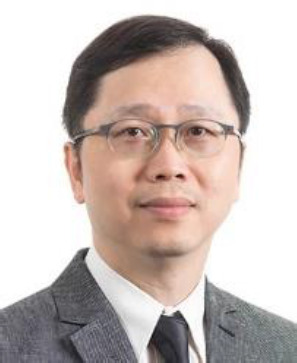


**Professor M.S. Wong** is a specialist in Prosthetics and Orthotics of the Department of Biomedical Engineering, The Hong Kong Polytechnic University. With his clinical and engineering training background, he has the educational vision of nurturing students with state-of-the-art professional knowledge and skills as well as all-rounded attributes, especially in positive attitude to tackle the uncertainties and challenges from this ever-changing world, and in social responsibility to serve the less-privileged people. His main research interests are scoliosis, spinal orthotics, prevention of fragility fractures, gait and posture analysis, CAD/CAM in prosthetics and orthotics, and prosthetics and orthotics outcome evaluations.

**Figure:**
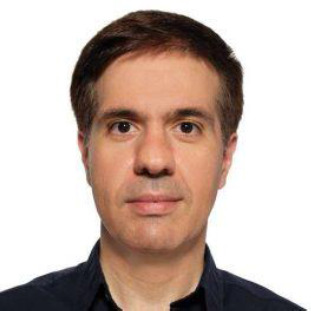


**Dr. Babak Hassan Beygi** is a prosthetist & orthotist targeting on enhancement of the clinical outcomes of applied orthotic and prosthetic appliances with his research and clinical background. His main interests include the conservative treatment of spinal deformities as well as application of CAD/CAM technology in orthotics & prosthetics.

## References

[1] Tan WS, Suwarno SR, An J, Chua CK, Fane AG, Chong TH. Comparison of solid, liquid and powder forms of 3D printing techniques in membrane spacer fabrication. J Membr Sci. 2017;537:283–96. DOI: 10.1016/j.memsci.2017.05.037

[2] The Evolution of 3D printing in orthotics and prosthetics: a game-changer in patient care [Internet]. TechMed 3D, 2023; [Cited 2023 Dec. 23]. Available from: https://techmed3d.com/blog/evolution-3d-printing-orthotics-prosthetics-patient-care/

[3] 3D printed prosthetic limbs allow patients to regain their mobility faster [Internet]. Alcam Medical Orthotics and Prosthetics, 2022; [Cited 2023 Dec. 23]. Available from: https://alcammedical.com/3d-printed-prosthetic-limbs/

[4] Schwartz DA, Schofield KA. Utilization of 3D printed orthoses for musculoskeletal conditions of the upper extremity: A systematic review. J Hand Ther. 2023;36(1):166–78. DOI: 10.1016/j.jht.2021.10.005 34819255

[5] Van Lieshout EMM, Verhofstad MHJ, Beens LM, Van Bekkum JJJ, Willemsen F, Janzing HMJ, et al. Personalized 3D-printed forearm braces as an alternative for a traditional plaster cast or splint; A systematic review. Injury. 2022;53 Suppl 3: S47–s52. DOI: 10.1016/j.injury.2022.07.020 35858868

[6] Oud T, Kerkum Y, de Groot P, Gijsbers H, Nollet F, Brehm MA. Production time and user satisfaction of 3-Dimensional printed orthoses for chronic hand conditions compared with conventional orthoses: A prospective case series. J Rehabil Med Clin Commun. 2021;4:1000048. DOI: 10.2340/20030711-1000048 33884150 PMC8054741

[7] Ten Kate J, Smit G, Breedveld P. 3D-printed upper limb prostheses: A review. Disabil Rehabil Assist Technol. 2017;12(3):300–14. DOI: 10.1080/17483107.2016.1253117 28152642

[8] Wong MS, Cheng JCY, Lo KH. A comparison of treatment effectiveness between the CAD/CAM method and the manual method for managing adolescent idiopathic scoliosis. Prosthet Orthot Int. 2005;29(1):105–11. DOI: 10.1080/17461550500069547 16180383

[9] Wong MS, Cheng JCY, Wong MW, So SF. A work study of the CAD/CAM method and conventional manual method in the fabrication of spinal orthoses for patients with adolescent idiopathic scoliosis. Prosthet Orthot Int. 2005;29(1):93–104. DOI: 10.1080/17461550500066782 16180382

[10] Lin Y, Cheung JPY, Chan CK, Wong SWF, Cheung KMC, Wong M, et al. A randomized controlled trial to evaluate the clinical effectiveness of 3D-printed orthosis in the management of adolescent idiopathic scoliosis. Spine. 2021. DOI: 10.1097/BRS.0000000000004202 34392277

